# A Broad Antiviral Strategy: Inhibitors of Human DHODH Pave the Way for Host-Targeting Antivirals against Emerging and Re-Emerging Viruses

**DOI:** 10.3390/v14050928

**Published:** 2022-04-28

**Authors:** Yucheng Zheng, Shiliang Li, Kun Song, Jiajie Ye, Wenkang Li, Yifan Zhong, Ziyan Feng, Simeng Liang, Zeng Cai, Ke Xu

**Affiliations:** 1 State Key Laboratory of Virology, College of Life Sciences, Wuhan University, Wuhan 430072, China; 2020102040034@whu.edu.cn (Y.Z.); kunsong@whu.edu.cn (K.S.); 2019202040053@whu.edu.cn (J.Y.); liwenkang@whu.edu.cn (W.L.); 2021202040011@whu.edu.cn (Y.Z.); 2015301060039@whu.edu.cn (S.L.); caiz@whu.edu.cn (Z.C.); 2 State Key Laboratory of New Drug Design, School of Pharmacy, East China University of Science and Technology, Shanghai 200237, China; shiliangli@ecust.edu.cn (S.L.); ziyan_fe@163.com (Z.F.); 3 Institute for Vaccine Research, Animal Biosafety Level 3 Laboratory at Center for Animal Experiments, Wuhan University, Wuhan 430072, China

**Keywords:** host-targeting antivirals (HTAs), DHODH inhibitors (DHODHi), pyrimidine synthesis, broad-spectrum antivirals

## Abstract

New strategies to rapidly develop broad-spectrum antiviral therapies are urgently required for emerging and re-emerging viruses. Host-targeting antivirals (HTAs) that target the universal host factors necessary for viral replication are the most promising approach, with broad-spectrum, foresighted function, and low resistance. We and others recently identified that host dihydroorotate dehydrogenase (DHODH) is one of the universal host factors essential for the replication of many acute-infectious viruses. DHODH is a rate-limiting enzyme catalyzing the fourth step in *de novo* pyrimidine synthesis. Therefore, it has also been developed as a therapeutic target for many diseases relying on cellular pyrimidine resources, such as cancers, autoimmune diseases, and viral or bacterial infections. Significantly, the successful use of DHODH inhibitors (DHODHi) against severe acute respiratory syndrome coronavirus 2 (SARS-CoV-2) infection further supports the application prospects. This review focuses on the advantages of HTAs and the antiviral effects of DHODHi with clinical applications. The multiple functions of DHODHi in inhibiting viral replication, stimulating ISGs expression, and suppressing cytokine storms make DHODHi a potent strategy against viral infection.

## 1. Introduction

In recent years, emerging or re-emerging viruses have appeared with increasing frequency, severely threatening global public health security and causing enormous economic losses [1]. At present, the World Health Organization has announced six international public health emergencies, the H1N1 influenza pandemic in 2009 [2], the polio epidemic in 2014 [3], the Ebola epidemic in West Africa in 2014 [4], the Zika epidemic in 2015–2016 [5], the Ebola epidemic in the Democratic Republic of Congo that began in 2018 (announced in July 2019) [6,7], and the severe acute respiratory syndrome coronavirus 2 (SARS-CoV-2) pneumonia pandemic that broke out at the end of 2019 [8]. In addition, Middle East respiratory syndrome coronavirus (MERS-CoV), SARS-CoV, dengue virus (DENV), Chikungunya virus, and other viruses cause epidemic infections worldwide [9,10,11,12]. These virus epidemics and pandemics remind us that a broad-spectrum antiviral must be prepared to combat the continual outbreaks of various viruses.

At present, most approved antivirals target viral proteins to inhibit specific steps in the viral infection cycle and are called direct-acting antivirals (DAAs). Taking SARS-CoV-2 as an example, in the rush of anti-SARS-CoV-2 drug development competition, remdesivir is highly expected. It targets viral RNA-dependent RNA polymerase (RdRp) to terminate viral RNA chain elongation by competing with the natural substrate of RdRp, adenosine triphosphate [13,14]. Unfortunately, although remdesivir showed fair anti-SARS-CoV-2 activity in vitro, clinical results showed no significant benefit compared with placebo [15,16], probably because of three reasons. (1) Remdesivir needs to be converted into remdesivir-TP in vivo to function [17,18]. (2) Remdesivir is originally developed by targeting the Ebola virus (EBOV) RdRp [19], making it unlikely to have an equivalent impact on the SARS-CoV-2 RdRp because of the plasticity of the virus. (3) The proofreading function of SARS-CoV-2 nsp14 exonuclease limits the potential of remdesivir [20]. Similar to remdesivir, many DAAs have been proven to be clinically successful only against a certain kind of virus but not others. Overall, DAAs have some inherent disadvantages. (1) They have narrow-spectrum antiviral properties. Viral proteins usually share few structural similarities among different species or classes. (2) Drug resistance develops against DAAs. DAAs act directly on viral proteins, promoting mutagenesis during viral replication. (3) DAAs are expensive and inefficient. DAAs apply the “one bug, one drug” strategy so that the development of individual DAA to each virus is an expensive and inefficient process in the context of continually emerging viruses [21].

Viruses are obligate parasites that rely entirely on the internal host environment to produce progeny viral particles. Therefore, viruses usually need to hijack the host machinery to replicate. For this reason, host-targeting antivirals (HTAs), which target the host factors required for viral infection, represent a broad-spectrum antiviral strategy. Moreover, the treatment of acute viral infections requires only a few days, greatly facilitating tolerance of the relative toxicity from the targeted host pathway. Compared with DAAs, HTAs have advantages. (1) HTAs show broad-spectrum antiviral activities because viruses use many of the same host proteins to replicate. (2) HTAs may also be effective against future emerging viruses. HTAs inhibit the host proteins essential for viral replication, which may also be effective against emerging viruses. (3) HTAs poorly induce the development of drug resistance. The host genetic material is double-stranded DNA with a lower mutation rate than RNA. Table 1 summarizes the host targets for antiviral treatment, such as dihydroorotate dehydrogenase (DHODH), chemokine receptor type 5, inosine monophosphate dehydrogenase, cyclophilins, eukaryotic initiation factor 2α, dihydrofolate reductase, and et al.

## 2. The Pyrimidine Synthesis Pathway Is a Reliable HTA Target

Pyrimidine is a heterocyclic compound and a vital component of cells [22]. Therefore, antivirals targeting the pyrimidine synthesis pathway may be effective and have a broad spectrum of activity. Pyrimidine participates in synthesizing not only nucleotides but also polysaccharides and phospholipids, which play an essential role in human metabolism. When cells become cancerous [23] or infected by pathogenic microorganisms [24,25], the overall metabolic activity and the demand for pyrimidines are increased compared with those in quiescent cells. Munger et al. researched human cytomegalovirus (HCMV)-infected human fibroblasts using liquid chromatography–tandem mass spectrometry and identified 167 differentially abundant metabolites. Among these metabolites, those related to *de novo* pyrimidine biosynthetic pathways, such as carbamoyl-aspartic acid, cytidine triphosphate, uridine triphosphate, and thymidine triphosphate, were significantly enriched compared to those in the uninfected group [26]. Another study by Consigli et al. found a similar phenomenon. The activity of aspartate transcarbamylase, which is essential for pyrimidine synthesis, was significantly increased in adenovirus type 5-infected HeLa cells [27]. These reports suggested that viruses would hijack the host pyrimidine synthesis pathway to benefit their replication.

**Figure 1:**
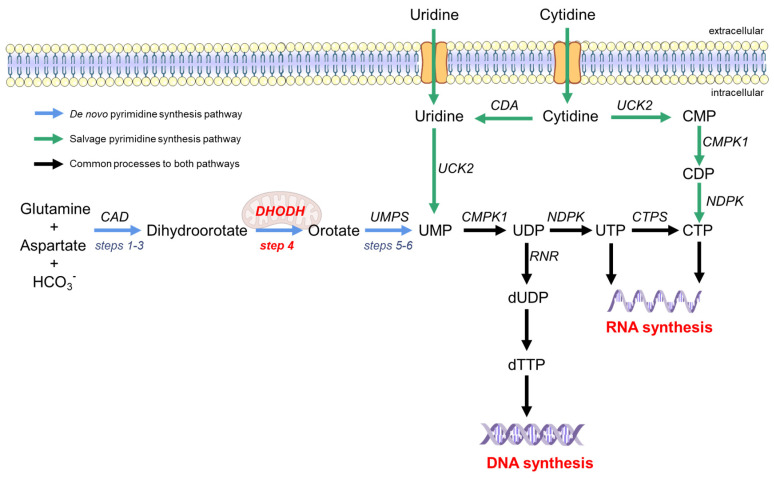
Pyrimidine synthesis pathway in humans. The *de novo* synthesis pathway of pyrimidine is represented by blue arrows, and the salvage pathway is represented by green arrows. The *de novo* synthesis pathway begins with dihydroorotate from glutamine and aspartate under the action of CAD multifunctional enzymes (steps 1–3). The mitochondrial inner membrane protein DHODH oxidizes DHO to produce orotate (step 4). Orotate is subsequently phosphorylated and produces UMP from the bifunctional enzyme UMPS (steps 5–6). In the salvage pathway, exogenous uridine and cytidine can be transformed into UMP and CTP, respectively. UDP is the raw material for DNA synthesis. CTP and UTP are the raw materials for RNA synthesis. CAD, carbamoyl phosphate synthetase, aspartate transcarbamoylase, and dihydroorotase; UMP, uridine monophosphate; DHODH, dihydroorotate dehydrogenase; UDP, uridine diphosphate; UTP, uridine triphosphate; CMP, cytidine monophosphate; CDP, cytidine diphosphate; CTP, cytidine triphosphate; CTPS, CTP synthase; dUDP, deoxy-UDP; dTTP, deoxythymidine triphosphate; UMPS, uridine monophosphate synthetase; CMPK, cytidine monophosphate kinase; NDPK, nucleoside-diphosphate kinase; CDA, cytidine deaminase; UCK, uridine or cytidine kinase; RR, ribonucleotide reductase.

There are two ways to synthesize pyrimidines in the human body, namely, the *de novo* biosynthesis and salvage pathways [25], as shown in Figure 1. The salvage pathway utilizes extracellular uridine or cytidine to resynthesize pyrimidine nucleotides through simple enzymatic reactions (green arrow in Figure 1). The salvage pathway is the primary source of quiescent or differentiated cells, but it is insufficient to provide the pyrimidine pool for highly proliferating and virus-infected cells [28,29]. Therefore, the *de novo* synthesis pathway is required for these cells. The *de novo* synthesis pathway provides a large pool of pyrimidine nucleotides by using simple precursor molecules (such as amino acids, CO_2_, and pentose phosphate) as substrates through a series of complex enzymatic reactions (blue arrow in Figure 1) [30]. Step 4 is the rate-limiting step in the *de novo* synthesis pathway, and dihydroorotate dehydrogenase (DHODH) is the only enzyme that oxidizes dihydroorotate (DHO) acid to orotate (ORO) [31].

## 3. The Essential Role of DHODH in the *De Novo* Pyrimidine Synthesis Pathway

The *de novo* pyrimidine synthesis pathway is divided into six steps (Figure 1) [30]. The first three steps proceed via the multifunctional CAD (carbamyl phosphate synthase, aspartate transcarbamylase, and dihydroorotate) enzymes catalyzing the conversion of L-glutamine, aspartic acid, and bicarbonate to dihydroorotate (DHO) (steps 1–3). Then, the mitochondrial membrane protein dihydroorotate dehydrogenase (DHODH) oxidizes DHO to ORO (step 4), which is the rate-limiting step of the *de novo* pyrimidine synthesis pathway [32]. ORO undergoes the action of orotate phosphoribosyltransferase and orotate 5′-monophosphate decarboxylase to generate uridine monophosphate (steps 5 and 6). In step 4, DHODH, as an oxidoreductase, removes two electrons from DHO and transfers them to flavin mononucleotide (FMN). FMN regeneration is necessary for sustained DHODH catalysis. For this reason, ubiquinone, the electron acceptor in the mitochondrial electron transport chain, is required to receive electrons from FMN to complete the catalytic cycle [30]. This specific role of DHODH, which is involved in the *de novo* pyrimidine synthesis and links this pathway to the electron transport chain of aerobic respiration, makes DHODH the most attractive drug target in the pyrimidine synthesis pathway.

**Figure 2:**
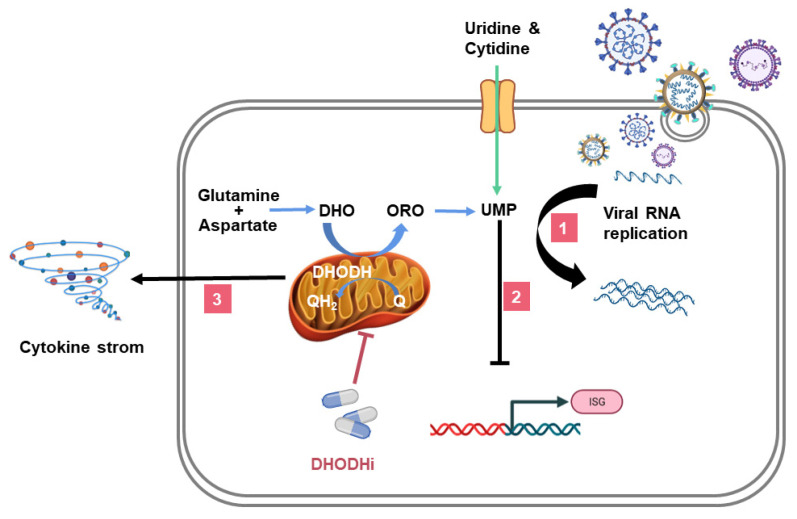
The role of DHODHi in viral infection. The triple mechanism of DHODHi is as follows: (1) DHODHi reduce the pyrimidine pool required for viral replication; (2) DHODHi activate ISGs expression; and (3) DHODHi suppress the inflammatory factor storm caused by the virus. The mechanisms by which human cells obtain pyrimidines: the *de novo* biosynthesis (blue arrow) and the salvage pathway (green arrow). UMP, uridine monophosphate; DHO, dihydroorotate; ORO, orotate; Q, ubiquinone; QH_2_, ubiquinol; ISG, interferon-stimulated gene.

**Figure 3:**
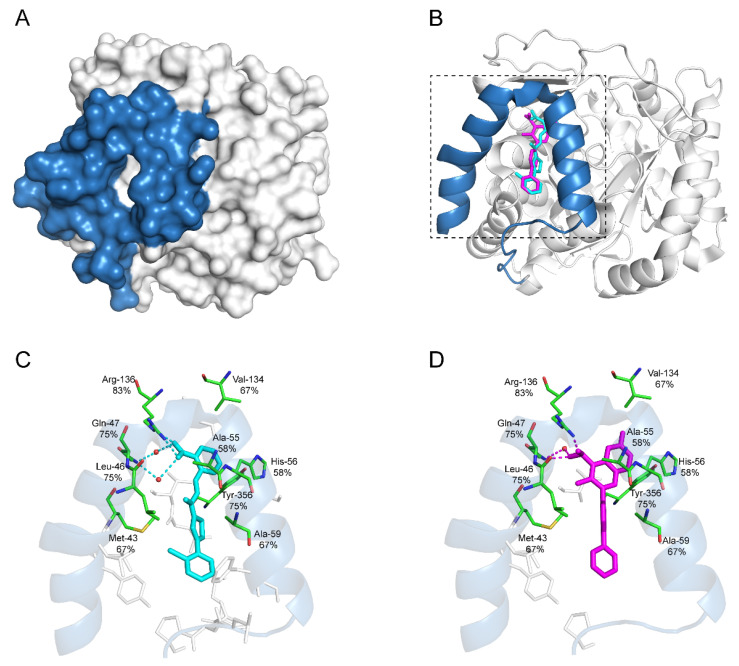
Recurring residues of DHODHi in ubiquinone-binding site. (**A**) 3D structure of human DHODH (PDB ID: 6M2B) with ubiquinone-binding site shown in blue. (**B**) Ribbon diagram of human DHODH in complex with S416 (cyan) and brequinar (purple). The C-terminal region (aa 78-395) is colored in white, and the N-terminal domain consisting of two α helices (binding sites for ubiquinone) is colored in blue. (**C**) Ribbon diagram of the DHODH ubiquinone-binding site in complex with S416 (cyan). S416 binds 9 recurring residues with 4 hydrogen bonds shown in the dashed line. (**D**) Ribbon diagram of the DHODH ubiquinone-binding site in complex with brequinar (purple). Brequinar binds 7 recurring residues with 3 hydrogen bonds shown in the dashed line. (**C**,**D**) The oxygen atom is marked in red, and the nitrogen atom is marked in blue. The water molecule is depicted as the red ball. Recurring binding residues are indicated as thin green rods, and the corresponding recurring frequencies among all the listed twelve drugs are marked underneath each amino acid. The other non-recurring binding residues specific to each drug are marked in thin grey rods.

DHODH inhibitors (DHODHi) have been used to treat malignant tumors, autoimmune diseases, viral or bacterial infections, parasitic diseases, and other diseases [23,31,33,34,35]. DHODHi inhibit viral infection by three mechanisms: (1) inhibiting viral replication (pathway 1 in Figure 2), (2) promoting interferon-stimulated genes (ISGs) expression (pathway 2 in Figure 2), and (3) regulating inflammation (pathway 3 in Figure 2). This article reviews the critical role of DHODH in the *de novo* pyrimidine synthesis pathway during viral infections, with examples of several DHODHi and their clinical applications.

## 4. DHODHi Inhibit the Virus Replication Cycle

Cytosine, thymine, and uridine are essential components of DNA and RNA. Therefore, viral genome replication requires the synthesis of large amounts of pyrimidines, which enables the broad-spectrum antiviral activity of DHODHi. Compared with DNA viruses, RNA viruses require the unique uridine monophosphate (particular nucleotide produced by DHODH) in their genomes instead of thymidine monophosphate, which suggests that RNA viruses are more sensitive to DHODH activity [36]. At present, increasing studies have found that DHODHi inhibit the replication of RNA viruses, especially from the early stage of the virus replication cycle. In IBRS-2 cells infected with 100 TCID_50_ of FMDV (O/MY98/BY/2010), administration of 300 μM teriflunomide at the early stage of infection (0–4 h after infection) significantly inhibited 99% of the 2B mRNA level and VP1 viral protein expression [32]. Similarly, in a Junin virus-infected Vero cell model (MOI = 0.1), 50 μM teriflunomide mainly inhibited viral replication in the early and middle stages (0–6 h after infection) [37]. Intriguingly, in Vero or A549 cell models infected with DENV serotype 2 (MOI = 2), brequinar inhibited not only the early and middle phases, but also the later phases (RNA synthesis, virion assembly, or release) of the viral replication cycle [38]. Therefore, DHODHi may have potent antiviral effects at all steps of RNA virus replication.

Although not as powerful as they are against RNA viruses, DHODHi are also reported to inhibit the replication of DNA viruses. For example, in HCMV (Towne strain)-infected human primary embryonic lung fibroblasts (HEL 299), FK778, an oral DHODHi, exhibited a potential antiviral effect with an EC_50_ of 1.97 μM [39]. In the A549 cell model infected with human adenovirus 5 (MOI = 5), the virus titers were reduced by 6 logs by compound A3. Vaccinia virus was also sensitive to compound A3 [35].

## 5. DHODHi Stimulate the Expression of ISGs

Lucas-Hourani et al. screened stimulators of the innate antiviral response and established a link between pyrimidine biosynthesis and ISGs expression [40]. They identified a DHODHi, DD264, which possessed ideal antiviral effects by enhancing ISGs expression. Moreover, supplementation with uridine abolished the amplification of ISGs expression by DD264. However, whether DHODHi induction of the expression of ISGs depends on the classic JAK-STAT pathway is not yet clear. Jin et al. used the JAK inhibitor CP-690550 to block the JAK-STAT pathway in Peste des petits ruminants virus (PPRV)-infected HEK293T cells. Surprisingly, the transcription of ISGs could still be upregulated by brequinar. This result indicated that the induction of ISGs by brequinar was independent of the JAK-STAT pathway [41]. In addition, an anti-influenza virus study suggested that leflunomide could still play an antiviral role after inhibiting the tyrosine phosphorylation of JAK1 and JAK3 [42]. In contrast, the antiviral activity of FA-613 relied on interferon-dependent ISGs stimulation [43]. It seems that different DHODHi activate ISGs expression by triggering different pathways.

## 6. DHODHi Inhibit the Production of Inflammatory Cytokines

For a long time, cytokines and chemokines have been considered to play essential roles in immunopathology during viral infection, because excessive virus-induced inflammation contributes to severe disease and death [44,45,46,47,48,49]. The DHODHi, such as leflunomide and teriflunomide, have been clinically used to treat autoimmune diseases and suppress cytokine production [50,51,52,53,54], they may also regulate excessive inflammation induced by viruses. We previously proved that the combination of S312 and oseltamivir vastly reduced the pathogenic inflammatory cytokine levels of IL6, MCP-1, IL5, KC/GRO (CXCL1), IL2, IFN-γ, IP-10, IL9, TNF-α, GM-CSF, EPO, IL12p70, MIP3α, and IL17A/F in influenza A virus-infected mice [36]. Similarly, it was reported that elevated inflammatory factor levels are positively correlated with the severity of COVID-19, such as those of IL-2, IL-6, IL-7, IL-10, G-CSF, MCP, MIP1α, IFN-γ, IP-10, and TNF-α [55,56,57,58,59,60]. Our unpublished data indicated that DHODHi could also regulate hyperinflammation reactions in severe SARS-CoV-2-infected animals by reducing pathogenic inflammatory cytokines levels. Although more research and clinical studies are expected to illustrate the immune-regulation role of DHODHi against SARS-CoV-2 infection, our clinical observation already showed that leflunomide could reduce lung inflammation and the serum C-reactive protein level in COVID-19 patients [61].

Moreover, DHODHi would offer dual effects in minimizing immune overreaction induced by viral infection. It is believed that SARS-CoV-2 induces lung damage in two stages [46,62]. The virus replicates in the lungs directly, causing lung tissue damage in the first stage. The second stage is characterized by the massive expression of cytokines and chemokines and the migration of immune cells to the lungs, resulting in an excessive inflammatory response [55,56,57,58,59,60,63]. The severity of tissue damage in the first stage determines the degree of inflammation in the second stage. Thus, DHODHi could act in both stages to reduce lung damage by limiting viral replication in the first stage [64] and further inhibit the overexpression of cytokines and chemokines from the residue tissue damage in the second stage [65].

## 7. DHODHi Applications in Antiviral Treatment

A variety of DHODHi have been proven to inhibit viral infection in vitro and in vivo [64]. Table 2 lists the antiviral activities and their current clinical applications of major DHODHi that have been approved or are in the experimental phase. Currently, all the human DHODHi target the ubiquinone-binding site in the N-terminal domain of DHODH (aa 30–68). Several recurring critical binding residues inside the ubiquinone-binding pocket are targeted repeatedly by different DHODHi, indicating the essentials of these residues for developing potent DHODHi (Figure 3).

### 7.1. Leflunomide and Teriflunomide

Leflunomide is a prodrug that can be metabolized to its active metabolite teriflunomide. Teriflunomide inhibit DHODH activity by noncompetitively binding to ubiquinone [66] at an IC_50_ value of ∼600 nM [23]. The FDA has approved leflunomide for the clinical treatment of rheumatoid arthritis and psoriatic arthritis and teriflunomide for the clinical treatment of multiple sclerosis [67]. Both leflunomide and teriflunomide have been proven to have various antiviral activities in vitro. They inhibit the replication of SARS-CoV-2, cytomegalovirus, herpesvirus, BK virus, Epstein–Barr virus, respiratory syncytial virus (RSV), and influenza virus [37,41,42,61,68,69,70,71,72]. In an anti-Junin virus study, when teriflunomide was used in combination with the DAA drug ribavirin, the antiviral activity was superior to single-drug treatment [37]. In influenza A virus (H5N1 or H1N1)-infected mouse models, leflunomide treatment mitigated weight loss, reduced viral load in the lung, and prolonged the survival time [42]. In the same study, teriflunomide inhibited the replication of the H5N1 virus by blocking the activity of Janus kinase 1 (JAK1) and JAK3. Two studies from the same group showed that the reduction in alveolar fluid clearance, a pathophysiologic sequelae post-RSV infection, could be prevented by leflunomide and teriflunomide. At the same time, the drug effects could be reversed by exogenous uridine [68,70].

Leflunomide and teriflunomide also showed strong anti-SARS-CoV-2 activity in vitro; in particular, the antiviral activity of teriflunomide was ~2.6-fold higher than that of favipiravir (a DAA that inhibits viral RdRp) [36]. In addition, leflunomide was tested in a clinical trial for COVID-19 therapy at the People’s Hospital of Wuhan University, China [61]. The results for compassionate use showed that the shedding time of patients taking leflunomide (median 5 days) was significantly shorter than that of control patients (median 11 days), with *p* = 0.046. Additionally, C-reactive protein level was reduced in leflunomide-treated patients, confirming the dual antiviral and anti-inflammatory functions of leflunomide. In addition, as a novel anti-SARS-CoV-2 drug, leflunomide research received a total grant (£1.5 million) from LifeArc, a well-known public welfare research institution in the field of global pharmaceutical innovation, on 29 May 2020 (https://www.lifearc.org/news/covid-19-information/covid-19-funding/defeat-covid-study/, accessed on 15 March 2022).

### 7.2. Brequinar

Brequinar is a more potent DHODHi (IC_50_ value of 10 nM for human DHODH) than leflunomide or teriflunomide [73]. The FDA has approved brequinar for rheumatoid arthritis and multiple sclerosis. Unlike leflunomide and teriflunomide, brequinar competitively binds to ubiquinone and disrupts the catalytic cycle of DHODH [66]. It was recently reported that brequinar possessed potential broad-spectrum antiviral activity against flaviviruses (West Nile virus, yellow fever virus, DENV, and Zika virus), western equine encephalitis virus, EBOV, influenza virus, enterovirus, and vesicular stomatitis virus in vitro [38,41,74,75,76,77]. Additionally, adding exogenous uridine could reverse the antiviral activity in vitro, indicating that the antiviral effect of brequinar may be attributed to affecting pyrimidine synthesis [38,41,74,75,76,77]. Furthermore, a study by Li et al. demonstrated that brequinar exhibited antiviral efficacy in mice challenged with 100 LD_50_ of FMDV [77]. Brequinar significantly prolonged the survival time of infected mice and provided a 25% protection rate at 5 dpi (the virus-infected mice all died within 60 h) [77]. For SARS-CoV-2, Xiong et al. demonstrated that brequinar showed excellent anti-SARS-CoV-2 effect with CC_50_ = 231.30 μM, EC_50_ = 0.123 μM, and SI = 1880.49 [36]. Schultz et al. also found that, in a model of wildtype BALB/c mice infected with the SARS-CoV-2 Beta strain, combined treatment of brequinar and molnupiravir significantly reduced viral titers and pathology compared to using monupiravir alone [78].

### 7.3. S312 and S416

The anti-influenza virus activity of leflunomide (EC_50_ > 25 μM) and teriflunomide (EC_50_ = 35.02 μM) was insufficient in vitro. Brequinar has excellent anti-influenza virus activity (EC_50_ = 0.241 μM) but high cytotoxicity (CC_50_ = 2.87 μM) [36]. Therefore, it is necessary to develop novel DHODHi with high efficiency and low toxicity. Our previous study screened 280,000 compounds by hierarchical structure analysis and identified two potent DHODHi, S312 and S416, with high efficiency and low toxicity. S312 and S416 have the same novel scaffold, and both are thiazole derivatives. The particular chemical structures endow them with unique binding characteristics in the ubiquinone-binding pocket of human DHODH. Furthermore, compared to S312, the additional methyl group of S416 could strengthen the binding affinity with the small hydrophobic subsite on DHODH through a stronger Van der Waals interaction. In addition, two water molecules participate in forming a water-bridged hydrogen bond network, which is favorable for the binding of the scaffold. The hydrazine group provides stability with a biologically active conformation. The hydrophobic interaction and charge-assisted hydrogen bond interactions in the hydrophilic region of the pocket lead to complementation with the tunnel-shaped ubiquinone-binding site [79,80]. The combination of water-mediated effects and conformational advantages make S312 and S416 highly effective DHODHi with IC_50_ values of 29.2 nM and 7.5 nM, respectively [36].

*In vitro* experiments have proven the broad-spectrum antiviral activity of S312 and S416, including against influenza A virus (H1N1, H3N2, and H9N2), Zika virus, EBOV, and SARS-CoV-2 [36]. It was worth noting that S312 (EC_50_ = 1.56 μM, SI = 101.41) and S416 (EC_50_ = 0.017 μM, SI = 10,505.88) showed excellent anti-SARS-CoV-2 efficacy in Vero cells. In vivo experiments in influenza-infected mice showed that S312 was superior to oseltamivir (the DAA targeting neuraminidase of influenza viruses) in treating the late infection phase and reducing cytokine and chemokine storms in influenza virus-infected mice because of its dual antiviral and immune regulation activities. In addition, combined with oseltamivir, S312 could confer an additional 16.7% survival in the severely late infection stage [36].

### 7.4. PTC299

PTC299 is an oral DHODHi with an IC_50_ value of 1 nM for human DHODH [81]. PTC299 has favorable drug properties targeting hematological tumors and normalizes vascular endothelial growth factor levels in cancer patients [82]. A study by Luban et al. demonstrated that PTC299 inhibited SARS-CoV-2 replication with little cytotoxicity in Vero E6 cells (CC_50_ > 10,000 nM, EC_50_ = 2.6 nM, SI > 3800). In addition, they also suggested that PTC299 had broad-spectrum antiviral activity in vitro against viruses such as EBOV, poliovirus, hepatitis C virus genotype 1b, and Rift Valley fever virus. Moreover, PTC299 also had a dual mechanism of inhibiting viral replication and reducing the production of inflammatory cytokines, such as interleukin (IL)-6, IL-17A, and IL-17F [65]. PTC299 is currently being evaluated in phase II/III study PTC299-VIR-015-COV19 (FITE19) to treat COVID-19 (https://clinicaltrials.gov/ct2/show/NCT04439071, accessed on 15 March 2022).

### 7.5. IMU-838

IMU-838 is another oral selective immunomodulator that inhibits the intracellular metabolism of activated immune cells by blocking DHODH activity at an IC_50_ value of 160 nM [83]. It has been proven that the active moiety of IMU-838, vidofludimus, exhibits broad-spectrum antiviral activity in vitro, including against SARS-CoV-2. Notably, in SARS-CoV-2-infected cells, the combination of IMU-838 and remdesivir (a DAA) almost entirely reduced viral yield, suggesting that the combination of a DHODHi and DAA was an effective antiviral strategy [84]. Currently, IMU-838 is being evaluated for its therapeutic effect against COVID-19 in phase II clinical trial of COVID-19 therapy (CALVID-1, NCT04379271) (https://clinicaltrials.gov/ct2/show/NCT04379271, accessed on 15 March 2022).

### 7.6. Compound A3

Hoffmann et al. screened approximately 61,600 inhibitors of influenza virus replication and identified compound A3 with low toxicity and high antiviral activity (CC_50_ = 268 μM, EC_50_ = 0.178 μM, SI = 1505) [35]. Moreover, they also suggested that compound A3 had broad-spectrum antiviral activity, including against retroviruses, RNA viruses, and DNA viruses. Compound A3, inhibiting human DHODH at an IC_50_ of 1.13 μM, was more effective in combination with ribavirin (an HTA, a guanosine analog) in anti-arenavirus studies, further demonstrating the therapeutic benefits of combining a DHODHi and another HTA [85].

### 7.7. FA-613

Cheung et al. screened 50,240 compounds targeting influenza virus nucleoprotein, and FA-613 was found to inhibit influenza A virus infection [43]. Their further study indicated that FA-613 had broad-spectrum antiviral efficacy, including against enterovirus A71, highly pathogenic influenza A virus (H5N1 and H7N9), RSV, SARS-CoV, MERS-CoV, and human rhinovirus A. In addition, FA-613 had almost no cytotoxicity at the effective antiviral concentration. When BALB/c mice were challenged with 3 LD_50_ of influenza A/HK/415742Md/2009 (H1N1), FA-613 treatment (2 mg/kg per day) for three days protected 30.7% of the mice from death [43]. Mechanismly, the antiviral effect of FA-613 was reversed by the addition of exogenous uridine or orotic acid, which suggested that FA-613 may target DHODH [43]. However, the direct DHODH inhibition by FA-613 is still wondering.

### 7.8. BAY2402234

BAY2402234 is a novel potent selective DHODHi with an IC_50_ of 1.2 nM [86]. Mathieu et al. screened 492 compounds inhibiting SARS-CoV-2 replication and found that BAY2402234 blocked almost 100% of SARS-CoV-2 particle production at 0.6 μM [87]. In addition, the combination of teriflunomide, IMU-838/vidofludimus, and BAY2402234 inhibited SARS-CoV-2 replication and reduced viral yield by at least two orders of magnitude in Vero E6 and Calu-3 cells infected with wildtype, the Alpha variant, and the Beta variant of SARS-CoV-2 [88].

### 7.9. MEDS433

MEDS433, a DHODHi with an IC_50_ of 1.2 nM for human DHODH, was developed using 2-hydroxypyrazolo [1,5-a] pyridine as an acidic scaffold [89]. Calistri et al. demonstrated that MEDS433 inhibited in vitro replication of HCoV-OC43 (EC_50_ = 0.012 μM), HCoV-229E (EC_50_ = 0.022 μM), and SARS-CoV-2 (EC_50_ = 0.063 μM in Vero E6, EC_50_ = 0.076 μM in Calu-3) at nanomolar range with low toxicity [90]. Luganinia et al. demonstrated that MEDS433 inhibited herpes simplex virus-1 and -2 in vitro (EC_50_~0.1 μM) and exhibited highly synergistic antiviral activity when combined with acyclovir (a DAA) in a checkerboard assay [91].

### 7.10. RYL-634

RYL-634 is another potent inhibitor targeting human DHODH with an IC_50_ of 60 nM [92]. RYL-634 exhibited excellent broad-spectrum antiviral activity in vitro against hepatitis C virus, DENV, Zika virus, chikungunya virus, enterovirus 71, human immunodeficiency virus, RSV, and influenza virus [92]. Recently, Gong et al. further illustrated that RYL-634 had a high antiviral activity (EC_50_ = 0.079 μM) in EBOV-infected Huh7 cells (MOI = 0.1) [93].

## 8. Conclusions and Prospects

DHODHi were initially used to treat cancers or autoimmune disorders and have gradually been applied to antiviral therapies. When viruses infect host cells, nucleotide biosynthesis flux increases, and the cytokine storm is also triggered [55,94]. The triple antiviral effects of DHODHi, including inhibiting viral replication, suppressing inflammation, and activating ISGs expression, make DHODHi excellent antivirals. The large-scale screening has also resulted in DHODHi becoming leading antiviral compounds [35,75,85,95]. Of note, leflunomide and brequinar have been associated with clinical side effects, such as gastrointestinal symptoms, thrombocytopenia, reversible alopecia areata, and elevated liver enzyme levels [96,97,98,99,100]. The off-target effects, not pyrimidine synthesis blockade, may be responsible for the side effects [31]. Fortunately, these side effects were reversed after stopping treatment [101].

Although most DHODHi exhibit broad-spectrum antiviral activity in vitro, several small-molecule DHODHi have failed to show significant therapeutic effects in animal models or clinically. The failure might be due to the narrow window of these molecules or the recovery of exogenous uridine from the host. Therefore, developing a more efficient DHODHi is a priority. Our previous research indicated the high target occupancy rate and low toxicity of S416, making S416 the most potent anti-SARS-CoV-2 candidate compound in vitro reported to date [36]. The potent antiviral activity of S416 (CC_50_ = 178.6 μM, EC_50_ = 0.017 μM, SI = 10,505.88) fully illustrates the great potential of DHODHi in the treatment of viral infections.

Moreover, the combination of an HTA and a DAA showed superior antiviral effects compared to that of a single drug, such as teriflunomide in combination with ribavirin [37], brequinar in combination with molnupiravir (a DAA that inhibits viral RdRp) [78], and S312 in combination with oseltamivir [36]. As the drug targets are distinct between an HTA and a DAA, a synergistic treatment by combining these drugs could block multiple steps and factors in the virus life cycle. Especially, DHODHi have several significant advantages when combined with DAAs. (1) Due to the rapid replication cycle of viruses, DAAs usually need to be applied shortly after infection because the targeted viral components will amplify exponentially during the illness duration. However, DHODHi target the host DHODH, which keeps a relatively stable level during infection. A combination of DHODHi and DAA would have a superimposing inhibitory effect throughout the disease course compared to a single drug alone. (2) DAAs directly target viruses but have no effects on the host’s inflammatory responses. DHODHi harbor dual functions of inhibiting both viral replication and excessive inflammation cytokine expressions by reducing the cellular pyrimidine pool. (3) The uptake or incorporation of the nucleoside analogs may be increased when pyrimidines are limiting [78]. Therefore, when DHODHi are used in combined with DAAs of nucleoside analogs, the incorporation efficiency of nucleoside analogs would be further increased. (4) DHODHi are effective to various viruses and viral variants regardless of mutagenesis, so combining with DAA could expand the targeted viral spectrum.

Targeting universal host factors necessary for viruses can finally achieve broad-spectrum antiviral effects. As a promising example, DHODHi, which can effectively reduce the pyrimidine pool for viral replication, stimulate the ISGs expression, and suppress the virus-induced cytokine storm, could serve as a broad antiviral strategy against emerging and re-emerging viruses.

## Figures and Tables

**Table 1:** Host targets and antiviral activities of host-targeting antivirals (HTAs).

Host Targets	Description of Host Targets	HTAs	Known Antiviral Effects
DHODH	The rate-limiting enzyme in the *de novo* pyrimidine synthesis pathway	Leflunomide, teriflunomide, and brequinar	Influenza virus, HBV, HCV, EBOV, DENV, SARS-CoV-2, HIV, and ZIKV
Chemokine receptors type 5	A G-protein coupled receptor, which is an HIV-1 co-receptor associated with CXCR4	Maraviroc, PF-232798, TAK-220, and INCB9471	HIV
Inosine monophosphate dehydrogenase	The rate-limiting enzyme in the *de novo* biosynthesis of guanine nucleotides	Ribavirin, mycophenolic acid, mycophenolate mofetil, and mizoribine	RSV, HCV, HBV, HCMV, EMCV, ZIKV, and EBOV
Cyclophilins	A peptidyl-prolyl isomerase, catalyzing the isomerization of peptide bonds from *trans* to *cis* form at proline residues to facilitate protein folding	Cyclosporin A, NIM811, and alisporivir	HCV
Eukaryotic initiation factor 2α	A eukaryotic initiation factor required for most eukaryotic translation initiation	Nitazoxanide, tizoxanide, and RM5061	Influenza virus, HBV, HCV, EBOV, DENV, JEV, HIV, and ZIKV
Dihydrofolate reductase	An enzyme converting dihydrofolate into tetrahydrofolate for the *de novo* synthesis of purines, thymidylic acid, and certain amino acids	Methotrexate, trimetrexate, and 1-aryl-4,6-diamino-1,2-dihydrotriazines	ZIKV, influenza virus, and RSV
α-Glucosidase	An enzyme catalyzing the hydrolysis of glycosidic bonds in complex sugars	NB-DNJ and Celgosivir	HIV, HCV, human coronavirus, influenza A virus, and DENV
Kinases	An enzyme that catalyzes the transfer of phosphate groups from high-energy, phosphate-donating molecules to specific substrates	Sunitinib and erlotinib	DENV and EBOV
Sodium taurocholate cotransporting polypeptide	A multiple transmembrane transporter involved in the circulation of bile acids, and served as a common receptor of HBV and HDV	Myrcludex B, CsA, ezetimibe, and ritonavir	HBV and HDV
Farnesoid X receptor	A nuclear bile acid receptor that regulates the expression of bile acid transporters	GW4064, WAY362450, fexaramine, and chenodeoxycholic acid	HBV
Diacylglycerol acyltransferases	An enzyme catalyzing the terminal step in triacylglycerol synthesis	pradigastat	HCV

DENV, dengue virus; EBOV, Ebola virus; EMCV, encephalomyocarditis virus; HBV, hepatitis B virus; HCV, hepatitis C virus; HCMV, human cytomegalovirus; HDV, hepatitis D virus; HIV, human immunodeficiency virus; JEV, Japanese encephalitis virus; RSV, respiratory syncytial virus; SARS-CoV-2, severe acute respiratory syndrome coronavirus 2; ZIKV, Zika virus.

**Table 2:** Ongoing research of DHODHi in antiviral infections.

DHODHi	Key Binding Site Residues	Molecular Structure	Antiviral Activities	Clinical Applications
Leflunomide	Tyr356, Met 43, His56, Ala55, Ala59, Pro364, Val134, Gln47, Arg136, Phe98	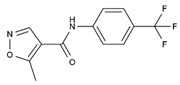	Influenza A virus (H1N1), ZIKV, EBOV, SARS-CoV-2, BK virus, DENV, porcine epidemic diarrhea virus, CMV, RSV, herpes simplex virus type 1, and HCMV	Phase I/II/III (SARS-CoV-2) Phase I (HIV) Phase II (BK virus)
Teriflunomide	Tyr356, Met 43, His56, Ala55, Ala59, Pro364, Val134, Arg136, Gln47, Phe98	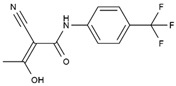	SARS-CoV-2, Human T-lymphotropic virus type-1, JUNV, influenza virus (H5N1), EBV, EV71, and HIV	Phase I/II (HTLV-1)
Brequinar	Arg136, Met 43, Gln47, Leu46, Leu42, His56, Tyr38, Pro326, Tyr356, Pro69, Val143, Val134	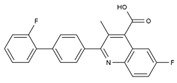	SARS-CoV-2 flaviviruses, alphavirus, rhabdovirus, influenza viruses, EV71, EV70, and Coxsackievirus B3	Phase I/II (SARS-CoV-2)
IMU838	Arg136, Met 43, Gln47, Leu46, Leu42, His56, Tyr38, Pro326, Tyr356, Pro69, Val143, Val134	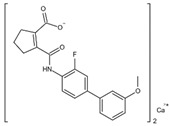	SARS-CoV-2, HCMV, HIV-1, and HCV	Phase II/III (SARS-CoV-2)
S416	Tyr38, Leu42, Met43, Leu46, Gln47, Pro52, Ala55, His56, Ala59, Phe62, Thr63, Leu67, Leu68, Pro69, Phe98, Met111, Val134, Arg136, Val143, Tyr356, Leu359, Thr360	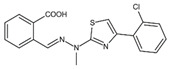	Influenza A virus (H1N1, H3N2, H9N2), ZIKV, EBOV, and SARS-CoV-2	——
S312	Tyr38, Leu42, Met43, Leu46, Gln47, Pro52, Ala55, His56, Ala59, Phe62, Thr63, Leu67, Leu68, Pro69, Phe98, Met111, Val134, Arg136, Val143, Tyr356, Leu359, Thr360	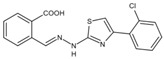	Influenza virus (H1N1, H3N2, H9N2), ZIKV, EBOV, and SARS-CoV-2	——
FA-613	Tyr356, Arg136, Ala55, Ala59, Leu 46, Thr360	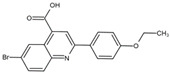	Influenza A virus (H5N1 and H7N9), EV-A71, RSV, human rhinovirus A, SARS-CoV, and MERS-CoV	——
PTC299	Tyr356, Phe98, Met111, Leu68, Pro364, Phe62, Met43, Leu58, Leu46, Leu50, Ala55, Arg136, His56, Ala59, Gln47, Val134, VAL143, Thr63	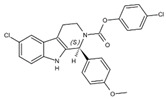	SARS-CoV-2, HCV, Poliovirus, EBOV, and Rift Valley Fever	——
Compound A3	Tyr356, Arg136, Ala55, Ala59, Leu46, Pro364, Phe336	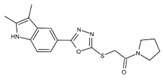	Influenza A virus (A/WSN/33), influenza B virus (B/Yamagata/88), Newcastle disease virus (La Sota), Sendai virus (SV52), Vesicular stomatitis virus, Sindbis virus, HCV, West Nile virus, DENV-1, NYVAC, hAd5, and HIV-1	——
BAY2402234	Thr63, Tyr38, Leu42, Met43, Leu46, Leu50, Leu58, Ala59, Phe62, Leu67, Leu68, Pro69, Met111, Leu359, Pro364, Thr360	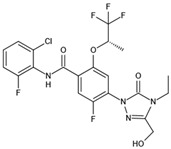	SARS-CoV-2	——
MEDS433	Gln47, Phe62, Arg136, Thr360	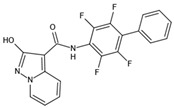	HCoV-OC43, HCoV-229E, SARS-CoV-2, and HSV	——
RYL-634	Tyr38, Leu42, Leu46, Gln47, Phe62, Leu67, Arg136	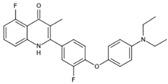	HCV, DENV, ZIKV, chikungunya virus, EV71, HIV, RSV, severe fever with thrombocytopenia syndrome virus, and influenza virus	——

CMV, cytomegalovirus; EBV, Epstein–Barr virus; EV70, enterovirus 70; EV71, enterovirus 71; hAd5, human adenovirus 5; HCoV-229E, human coronavirus 229E; HCoV-OC43, human coronavirus OC43; HSV, herpes simplex virus; JUNV, Junin virus; NYVAC, New York attenuated vaccinia virus; MERS-CoV, Middle East respiratory syndrome coronavirus; SARS-CoV, severe acute respiratory syndrome coronavirus. Data collected from Clinicaltrials.gov.

## Data Availability

Not applicable.
